# A Rare Case of Non-cirrhotic Acute Portal Vein Thrombosis With Gallbladder Infarction

**DOI:** 10.7759/cureus.78965

**Published:** 2025-02-13

**Authors:** Mitsunobu Toyosaki, Mao Tsukadaira, Yushi Matsuo, Junichi Sasaki

**Affiliations:** 1 Department of Emergency and Critical Care Medicine, Keio University School of Medicine, Tokyo, JPN; 2 Department of Emergency Medicine, Fussa Hospital, Fussa, JPN

**Keywords:** diagnostic challange, gallbladder ischemia, liver and gallbladder disease, portal vein thrombosis (pvt), potential pitfall for misdiagnosis, rare case

## Abstract

Non-cirrhotic portal vein thrombosis (PVT) is rare, and early initiation of anticoagulation therapy is crucial for recanalization and preventing complications.

A man in his 50s presented to the emergency department with acute back pain. His vital signs and laboratory results were normal, showing no signs of infection. An initial computed tomography (CT) scan with intravenous contrast in the arterial phase showed no abnormalities. However, on the third day, a CT scan in the late phase confirmed PVT in the left branch, along with gallbladder infarction.

This case highlights the limitations of arterial-phase CT in diagnosing acute PVT, which often extends to the splenic vein or superior mesenteric venous arches and may lead to intestinal infarction, although gallbladder infarctions remain rare.

## Introduction

Portal vein thrombosis (PVT) is common in patients with cirrhosis [1], but non-cirrhotic PVT is relatively rare. PVT is classified as recent or chronic, based on whether the thrombus has been present for more than six months. Recent PVT symptoms vary depending on the timing and extent of venous occlusion. “Acute” PVT refers to the recent formation of a thrombus within the portal vein and/or right or left branches [2]. Patients with acute PVT may complain of abdominal pain, whereas those with slowly progressing PVT are asymptomatic. PVT is generally diagnosed using computed tomography (CT) with intravenous contrast [2]. Anticoagulation therapy is crucial for recanalization and thrombosis prevention. Intestinal infarction, resulting from thrombotic progression to the superior mesenteric venous arches, is the most concerning immediate complication and can be fatal [3]. PVT can extend to the splenic veins, but progression to the gallbladder veins is rare and has not been previously reported. Herein, we present a case of acute non-cirrhotic symptomatic PVT with gallbladder infarction.

## Case presentation

A man in his 50s presented to the emergency department with the following vital signs: alert and oriented with a Glasgow Coma Scale score of 15, heart rate of 69 beats per minute (bpm), blood pressure of 98/61 mmHg, respiratory rate of 18 bpm, oxygen saturation of 99% on room air, pupils 3(+)/3(+), and body temperature of 35.8 ℃. The patient had a history of diabetes, but no known history of infection, cirrhosis, or thrombotic disorders. On the day of admission, while engaged in his usual activities, he suddenly experienced back pain. As the pain neither improved nor worsened, emergency services were contacted. Costovertebral angle tenderness was not confirmed, and no neurological abnormalities were observed. His abdomen was soft and flat, bowel sounds were intact, and exacerbation by compression or tapping was not confirmed. The intensity of abdominal pain was 10/10 on the visual analog scale. Increased white blood cell count (16,900/µL), no changes in inflammatory response (C-reactive protein, 0.16 mg/dL), hepatobiliary enzyme levels (aspartate aminotransferase (AST) = 21 U/L; alanine transaminase (ALT) = 21 U/L; lactate dehydrogenase (LDH) = 213 U/L; alkaline phosphatase (ALP) = 78 U/L; and bilirubin blood test (T-Bil) = 0.51 mg/dL), and coagulopathy (activated partial thromboplastin time (APTT) = 25.6 s; prothrombin time (PT), 123.9% and 11.2 s; and PT-international normalized ratio (INR) = 0.89) were confirmed in the laboratory data (Table 1).

**Table 1:** Laboratory data. CRP: C-reactive protein, Cl: chloride, Ca: calcium, Na: sodium, K: potassium, AST: aspartate aminotransferase, ALT: alanine transaminase, LDH: lactate dehydrogenase, ALP: alkaline phosphatase, γ-GTP: γ-glutamyl transpeptidase, CK: creatine kinase, BUN: blood urea nitrogen, CRE: creatinine, HbA1c: hemoglobin A1c, PCT: procalcitonin, PT: prothrombin time, APTT: activated partial thromboplastin time, INR: international normalized ratio, HBs-Ag: hepatitis B surface antigen, HCV: hepatitis C virus.

Variable	On the first day	On the third day	Normal range
Total protein (g/dL)	8.2	6.9	6.7–8.3
Albumin (g/dL)	5.1	3.7	3.9–4.9
Total bilirubin (mg/dL)	0.51	0.9	0.20–1.20
Direct bilirubin (mg/dL)	0.18	–	0.00–0.40
AST (U/L)	21	61	8–38
ALT (U/L)	21	72	4–44
LDH (U/L)	213	364	106–211
ALP (U/l)	78	78	38–113
γ-GTP (U/L)	–	39	16–73
CK (U/L)	291	540	56–244
BUN (mg/dL)	12.9	27.3	5.0–20.0
CRE (mg/dL)	0.73	0.9	0.50–1.30
Glucose (mg/dL)	167	158	60–110
HbA1c (%)	7.7	–	4.6-6.2
Na (mmol/L)	140.1	137	135–147
K (mmol/L)	3.74	3.99	3.5–5.5
Cl (mmol/L)	100.4	99.5	98–108
Ca (mg/dL)	9.8	8.9	8.8–10.2
CRP (mg/dL)	0.16	41.05	0–0.3
PCT (ng/mL)	–	14.64	0.00–0.05
White blood cell (10^2^/µL)	169	177	35–80
Red blood cell (10^4^/µL)	498	522	370–500
Hemoglobin (g/dL)	14.7	15.1	11.3–15.0
Hematocrit (%)	43	45.3	33.0–44.0
Platelet (10^4^/µL)	30.4	23.1	15.0–35.0
APTT (s)	25.6	49.3	25.0–40.0
PT (%)	123.9	70.6	70.0–130.0
PT (s)	11.2	15.2	–
PT-INR	0.89	1.22	0.90–1.10
D-dimer (µg/mL)	1	5.3	0.0–1.0
HBs-Ag index (IU/mL)	0	–	0.00–0.05
HCV index (S/CO)	0.07	–	0.00–1.00

**Figure 1:**
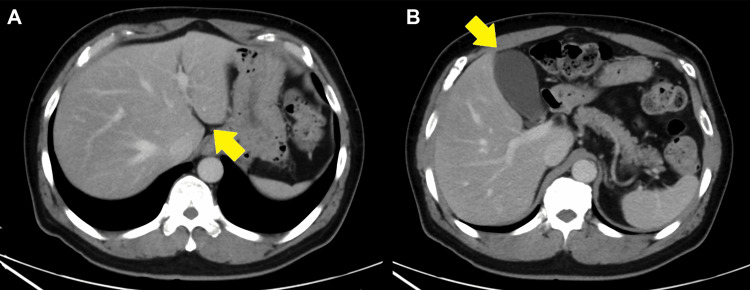
Computed tomography scan with arterial phase contrast on the first day. (A) No abnormalities in the arteries, portal veins, or bloodstreams of other organs were observed, although the portal veins were not clearly enhanced. (B) No gallbladder infarction, thickening of the gallbladder wall, or discontinuation of the gallbladder artery was observed.

A chest and pelvic CT scan with intravenous contrast in the arterial phase was performed to diagnose potential vascular emergencies. No marked abnormalities were detected, and radiology specialists noted only fatty liver (Figure 1).

**Figure 2:**
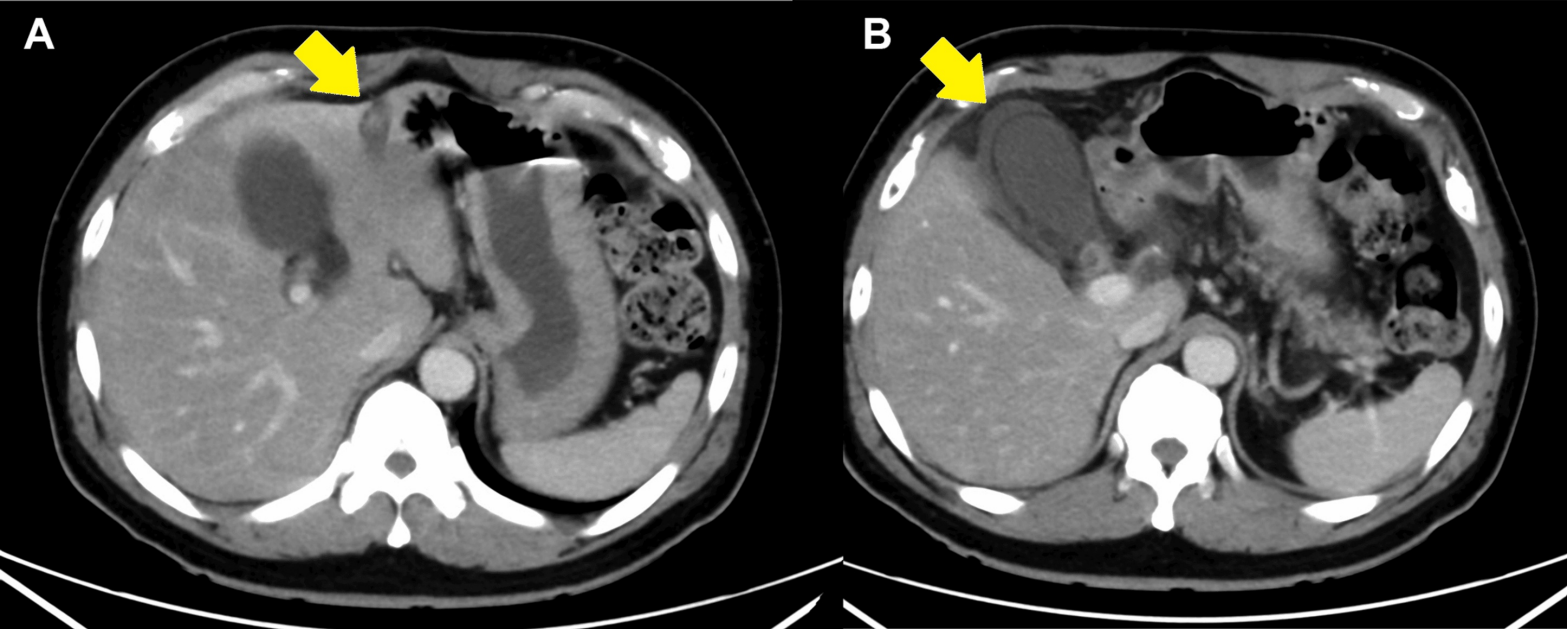
Computed tomography scan with late phase contrast on the third day. (A) Portal vein thrombosis in the left branch and no partial enhancement in the liver were observed. (B) Gallbladder infarction with no enhancement nor thickening of the gallbladder wall was observed.

Magnetic resonance imaging of the spine without contrast was performed, without remarkable findings. After acetaminophen injection, the patient’s back pain gradually improved, although its cause remained unresolved. The patient was admitted for observation. On the second day, a gastric fiber test was performed to exclude gastric anisakiasis, without abnormal findings. On the third day, fever (38.9 ℃), increased white blood cell count (17700/µL), inflammatory response (C-reactive protein, 41.05 mg/dL), elevated hepatic enzyme levels (AST = 61 U/L; ALT = 72 U/L; LDH = 364 U/L; ALP = 78 U/L; and T-Bil = 0.9 mg/dL), and coagulopathy (APTT = 49.3 s; PT, 70.6% and 15.2 s; and PT-INR = 1.22) were observed in the laboratory data (Table 1). Therefore, another CT scan with intravenous contrast in the late phase (not the portal phase) was performed, and PVT in the left branch with gallbladder infarction was confirmed (Figure 2).

The patient was transferred to a tertiary care facility for advanced care, where anticoagulation was initiated. Recanalization was not performed during admission. The patient was discharged and followed up as an outpatient. Cholecystectomy was deferred until recanalization was achieved.

## Discussion

Frequency of non-cirrhotic PVT

Non-cirrhotic PVT is infrequent, and accurate data on its frequency is scarce. According to one study, the incidence rate of PVT (35% in patients with cirrhosis) was 3.8 per 100,000 inhabitants in men and 1.7 per 100,000 inhabitants in women [4]. These frequencies reveal that non-cirrhotic PVT is rare, especially in individuals with back pain (not abdominal pain). Patients with PVT who visit the emergency room do not routinely have superior ranks for differential diagnoses. In this case report, no other risk factors for PVT were identified, and differential diagnoses other than PVT were considered, including vascular emergency diseases, acute aortic dissection, and thrombosis of the superior mesenteric artery.

Diagnosis of acute PVT

According to the European Association for the Study of the Liver clinical practice guidelines, the diagnostic guidance for PVT recommends ultrasound (diagnosis accuracy of 88%-98%) as the first screening modality and CT with venous contrast for a definitive diagnosis. Furthermore, imaging at the correct time (portal phase) is mandatory because the late arterial phase is not optimal for the diagnosis of PVT [2]. Some experts recommend the “portal phase” 80 s after contrast injection as suitable for diagnosis [5]. However, no study has yet assessed the diagnostic accuracy of CT. In our patient, no ultrasound screening was implemented, since we decided to implement an immediate CT scan to exclude fatal vascular diseases as mentioned above. Therefore, the initial CT scan with intravenous contrast captured only the arterial phase without accurately imaging the “portal phase.” This likely contributed to the diagnostic failure of PVT. A well-timed CT scan, ideally performed early in the diagnostic process, would aid in visualizing the portal vein and detecting any thrombosis or occlusion. Additionally, on the initial CT, the gallbladder wall was enhanced, and no shadow defects nor abnormalities in the gallbladder appearance (gallbladder wall thickening and debris) were observed. This finding suggests that no gallbladder infarctions occurred, indicating that the gallbladder infarction progressed following PVT of the left branch.

Anticoagulation therapy and complications of acute PVT: gallbladder infarction

Acute PVT often progresses to the splenic vein or the superior mesenteric venous arch and causes intestinal infarction. One study prospectively followed up patients with non-cirrhotic acute PVT; anticoagulation was administered to 95 (93%) of the 102 patients, and the splenic and superior mesenteric veins were obstructed in 41 (43%) and 55 (58%) of the 95 patients receiving anticoagulants at diagnosis, respectively. Moreover, among the 102 patients, 22 had local risk factors such as infected or bleeding disorders, including cholecystitis or cholangitis. Of the 95 patients who received anticoagulation therapy, 83 exhibited obstruction of the portal vein or its two branches. Recanalization was achieved in 38% of these patients within one year. Intestinal infarction occurred in two patients, and two patients died from causes unrelated to PVT and the treatment [6]. Obstruction of the gallbladder veins or gallbladder infarctions has not been reported, and biliary complications can also occur in patients undergoing PVT. Portal biliopathy, the abnormal appearance of intrahepatic and extrahepatic bile ducts and the gallbladder wall, is observed in patients with portal hypertension caused by extrahepatic PVT [7]. Portal biliopathy is the result of the formation of a cavernoma by collateral veins (developing a few weeks after PVT) and is not an acute change. Moreover, the pathological conditions of portal biliopathy are biliary obstructions due to compression of the bile duct or gallbladders and not thrombosis of the gallbladder veins. In our patient, gallbladder infarction was considered to have occurred because of the progression of thrombosis. This is because the interval between the first symptom and the gallbladder infarction diagnosis was only 2 days. Furthermore, having not initiated anticoagulation therapies because PVT was not diagnosed may have influenced the development of gallbladder infarction. Noteworthy, as this was only one case, the accidental combination of acute PVT and gallbladder infarction could not be excluded.

## Conclusions

We present a rare, nonfatal case of acute non-cirrhotic PVT with gallbladder infarction. In this case, the patient did not have cirrhosis, and the diagnosis was delayed because PVT was not initially considered in the differential diagnosis. Accurate imaging in the "portal phase" is essential for diagnosing PVT. Therefore, in patients with acute abdominal (or back) pain, CT should include the portal phase, even in the absence of cirrhosis. Additionally, gallbladder infarction should be considered a rare but possible complication of acute PVT.
